# *Hematodinium* sp. infection alters hemolymph bacteriome composition and function in shore crabs, *Carcinus maenas*

**DOI:** 10.1080/21505594.2026.2709259

**Published:** 2026-07-26

**Authors:** Jessica E. Thomas, Miguel Lurgi, Charlotte E. Davies, Andrew F. Rowley, Christopher J. Coates

**Affiliations:** a Department of Biosciences, Faculty of Science and Engineering, Swansea University, Swansea, UK; b Zoology and Ryan Institute, School of Natural Sciences, University of Galway, Galway, Ireland

**Keywords:** 16S rRNA metabarcoding, dysbiosis, host-pathogen interactions, innate immunity, marine disease, pathobiome

## Abstract

*Hematodinium* sp. is a parasitic dinoflagellate that infects and causes disease in a large fraction of decapod crustaceans in the Northern Hemisphere. The shore crab *Carcinus maenas* is susceptible to this parasite in its native range of European coastlines and along its invasive fronts. The parasite enters the host from the surrounding water, and fueled by the hemolymph (blood) resources, proliferates until host tissues are overrun. To improve our knowledge of the shore crab-*Hematodinium* pathosystem, we profiled the hemolymph microbiome of control and *Hematodinium*-positive crabs with low (early clinical) and high (late clinical) parasite levels through bacterial 16S rRNA V3-V4 meta-barcoding (*n* = 64). Concurrently, we assessed hemolymph condition through quantification of circulating hemocytes (immune cells) and bacterial colony forming units. Progression of *Hematodinium* sp. infection coincided with gradual changes in bacterial community composition. These changes were driven by shifts in relative abundances of specific taxa, e.g. *Corynebacterium, Flavobacterium*. Furthermore, alterations in bacterial communities were accompanied by marked shifts in metabolic signatures from stress and virulence dominated (including biofilm formation) to catabolism, peptide transport, and tissue putrefaction at the terminal stage of parasitemia. We present the first account of microbiome dynamics in healthy shore crabs and those overcome by *Hematodinium* sp. Our experiments reveal a clear link between bacterial community structure-function and the advancing disease state.

## Introduction

Fouling and infectious disease represent substantial hazards to the sustainability of shellfish capture production (fisheries) and aquaculture intensification [1–3]. A myriad of known pathogenic agents target decapod crustaceans, notably bacteria (e.g. *Vibrio* spp [4]), viruses (e.g. White Spot Syndrome Virus [5]), Microsporidia (reviewed by [6]), and macro-parasites (e.g. sacculinids [7]). Considerably less attention has been given to dinoflagellate-related pathologies [8] – an exception being parasites of the genus *Hematodinium* [9]. *Hematodinium* spp. epizootics, host diversity, and geographic reach, as well as limited histopathologic deterioration, have been documented clearly and repeatedly (reviewed by [9–12]). For example, the *Nephrops-Hematodinium* sp. pathosystem in the Clyde Sea Area of Scotland has been surveyed for >30 years [13,14], demonstrating that this pathogen is enzootic and temporally synchronous (peak disease in Spring/Summer). With the ever increasing number of studies documenting *Hematodinium* spp. alongside the spread of invasive decapods [15–17], there is a pressing need to gain further knowledge on those animals that are susceptible or resistant to the parasite. This is especially important if we seek to identify the causes behind some marine environments lacking *Hematodinium* spp. outbreaks and to design mitigation measures for where they do occur (e.g. [12,18]).

An area of weakness in this field is our limited grasp of host-pathogen interactions at the cellular and molecular levels, notably the immune interface [19,20] and the host microbiome(s) [21]. Recent experiments profiling hemolymph microvesicle traffic [22,23] and a shift in focus to omics-based approaches [24] have provided much needed insight into apparent immune-dysregulation. Reduced numbers of hemocytes in the hemolymph (haematocytopenia) and depleted protein levels (hypoproteinemia) promote a coagulopathic state [19,25,26], and, susceptibility to opportunistic infections at the terminal stage of disease when host mortality is assured [27]. Earlier suggestions by Field and Appleton [28] that *Hematodinium* spp. interfere with hematopoiesis have not been validated, though *Hematodinium*-derived miRNAs were linked to altered hemocyte phagocytosis of bacteria in the *H. perezi*-Chinese swimming crab (*Portunus trituberculatus*) pathosystem [23].

Once inside the host, *Hematodinium* spp. proliferate unchecked in the hemolymph and visceral organs, such as the gonads, hepatopancreas, gut, and gills. As the infection progresses, the host’s health can deteriorate in several ways including altered metabolism (e.g. respiratory capacity [29]), behavior [30], physical appearance (e.g. exoskeleton discoloration [31]), and immune responsiveness (e.g. [32]). We caution the reader that not all *Hematodinium*-crustacean interactions display the same clinical profile, rates of decline, or survival outcomes. Occasionally, co-infections of *Hematodinium* spp. and various pathogens are reported, such as yeast-like fungi in velvet swimming crabs (*Necora puber*, [33]) and brown crabs (*Cancer pagurus* [34]). While it is logical to assume that *Hematodinium*-afflicted crustaceans would be weakened, and therefore more vulnerable to co-infections, Davies et al. [20] reported that shore crabs (in their native range) testing positive for sub-patent or patent *Hematodinium* sp. infection were no more likely to be co-infected with other well-known parasites/pathogens (e.g. haplosporidians) than those lacking *Hematodinium.*

Host-associated communities of bacteria, archaea, protozoa, fungi, and viruses (i.e. the microbiome) display distinct physico-chemical properties, temporal and spatial plasticity, sensitivity to external (environmental) factors, and importantly, inform the diet and condition of their host (reviewed by Bass et al. [35] in the context of the “pathobiome”). Whether the host is a prawn, finfish, plant, or human, the inhabitant microbiota are inextricably linked to health and disease [36–38]. Relatively few studies have looked into crustacean microbiomes (e.g. *Homarus gammarus*, Holt et al. [39]; *Necora puber*, Martin et al. [40]), and those covering parasite-driven dysbiosis are even fewer. Flatback mud crabs, *Eurypanopeus depressus*, infected with a barnacle parasite, *Loxothylacus panopaei*, housed microbial populations of host and parasite origin, whereas the microbiomes of uninfected crabs were more distinct and variable [41]. Martin et al. [21] first reported on microbiome-level changes in a *Hematodinium*-infected crustacean, the Norway lobster *N. norvegicus*. The establishment of infection in the lobster, defined by sub-clinical *Hematodinium* sp. diagnosis (PCR-positive only), coincided with the most obvious shift in bacteria, i.e. a reduction in hemolymph bacterial richness compared to control animals. The initial dysbiosis persisted into the later (clinical) stages of infection.

To disentangle the drivers of host-associated microbiome dynamics across healthy and parasitized states, we aimed to: (1) profile the hemolymph microbiome of the globally invasive shore crab *C. maenas* using high throughput sequencing targeting the bacterial 16S rRNA V3-V4 region, (2) differentiate putative host/environmental traits associated with bacterial profiles, and (3) interrogate the bacteriome structure and function of parasitized (*Hematodinium* sp. positive) crabs.

## Materials and methods

### Crab samples

In a targeted disease survey, 1,191 shore crabs (*Carcinus maenas*) were collected across two sites in Swansea Bay (Wales, UK) – an open water pier (Mumbles; 51°34′8.958″N, 3°58′33.297″W) and semi-closed dock (Prince of Wales; 51°37′8.76″N, 3°55′36.84″W) – between November 2017 and October 2018 using baited Swedish crayfish traps (pots) that were immersed on site for 24 hours (as detailed in [42]). Living crabs, *n* = ~50 per site per month, were bagged individually, placed on ice, and transported back to the laboratory (<2 hours) where they were processed immediately. Biometric data recorded for each crab included carapace width (using Vernier calipers), biological sex, missing limbs, shell damage and/or fractures, and the presence of fouling agents (epibionts) or ectoparasites. Approximately 350 µL of hemolymph per crab were withdrawn through the soft intersegmental surface of a walking limb (surface sterilized with 70% [v/v] ethanol) using a 22/23-gauge hypodermic needle attached to a sterile syringe. The appearance of hemolymph was noted as milky (opaque, potential marker of sepsis) or clear (translucent). Sub-samples of hemolymph were either inspected fresh, fixed for cell/parasite counts or archived (100 µL at − 70 to −80°C) for later gDNA extraction and PCR-based screening for *Hematodinium* sp. (see *Hematodinium* sp. diagnoses and host hemolymph parameters).

Following hemolymph extraction, every crab was dispatched by chilling on ice and direct intrahaemocoelic injection of Davidson’s seawater fixative (containing ~10% v/v formalin [42]). Three gills and three hepatopancreas/gonad biopsies (~0.5 cm^3^) were excised from each dissected crab and submerged in Davidson’s seawater fixative for 24 hours prior to their storage in 70% (v/v) ethanol.

### Hematodinium sp. diagnoses and host hemolymph parameters

For each crab, a 25 µL subsample of fresh hemolymph was placed on a glass slide and left to settle at room temperature (~5 minutes) prior to microscopy-based inspection for the putative presence of *Hematodinium* spp. using phase contrast settings (BX41 Olympus microscope, Tokyo, Japan). A further 25 µL of hemolymph was fixed 1:1 with 5% formaldehyde (containing 3% [w/v] NaCl) for enumerating circulating hemocyte numbers – and potential *Hematodinium* sp. trophonts – using an improved Neubauer hemocytometer. Total numbers of host immune cells (hemocytes) and *Hematodinium* sp. morphotypes per mL hemolymph were calculated and the ratio of hemocytes to *Hematodinium* sp. was established.

To account for sub-patent levels of *Hematodinium* sp., ~100 µL of frozen-thawed hemolymph was tested using PCR by following the protocols of Davies et al. [42]. Briefly, gDNA was isolated using the Blood and Tissue Kit from Qiagen (Hilden, Germany), checked for contaminants (e.g. salt, protein) using a nanophotometer, and quantified using an Invitrogen Qubit® dsDNA High Sensitivity Assay and fluorimeter (Qubit® Fluorometer; California, USA). Each PCR reaction (25 µL) consisted of 50 - 200 ng DNA, 2x Master Mix from New England Biolabs (Ipswitch, USA) and an equimolar concentration of oligonucleotide primers synthesized by Eurofins (Ebersberg, Germany). Two primer sets targeting the 18S rRNA region (First, Hemat-F-1487 and Hemat-R-1654; Second, 18SF2 and Hem3R) were used to establish the presence/absence of *Hematodinium*, and species identity, respectively (see Supplementary Table 1). Positive samples – PCR amplicons visualized using 2% agarose/TBE with GreenSafe stain (NZYTech, Lisboa, Portugal) – were re-amplified, cleaned-up using the HT ExoSAP-IT™ Fast high-throughput PCR product kit (Thermo Fisher Scientific, Altrincham, UK), and sequenced using Sanger’s method (forward and reverse reads were performed by Eurofins). PCR-based probing yielded 162 *Hematodinium*-positive signals among crabs (13.6% prevalence); sequences are available in GenBank (MN057783–MN057918). These crab-derived samples were size, sex and site-matched to a further 162 *Hematodinium*-negative controls, and all 324 crabs were profiled further via histology using the previously archived gill and hepatopancreas/gonad samples (following the H&E protocol of [20,42]). Histology grades 0 to 4 were used previously by Smith et al. [43] for *Hematodinium*-infected brown crabs (*Cancer pagurus)* and by Davies et al. [42] for *Hematodinium*-infected shore crabs, in which 0 represents no visible parasites in tissue micrographs and 4 denotes tissue replete with parasites.

Additionally, 100 µL fresh/unaltered hemolymph per crab was mixed 1:1 with sterile saline (3% NaCl, w/v), plated onto a general growth medium (tryptone soya agar with 2% NaCl added) and incubated at 25°C for up to 48 hours. Bacterial colony forming units (CFUs) were enumerated and presented as CFUs per mL hemolymph. Two technical replicates were performed per crab sample.

**Figure 1.**
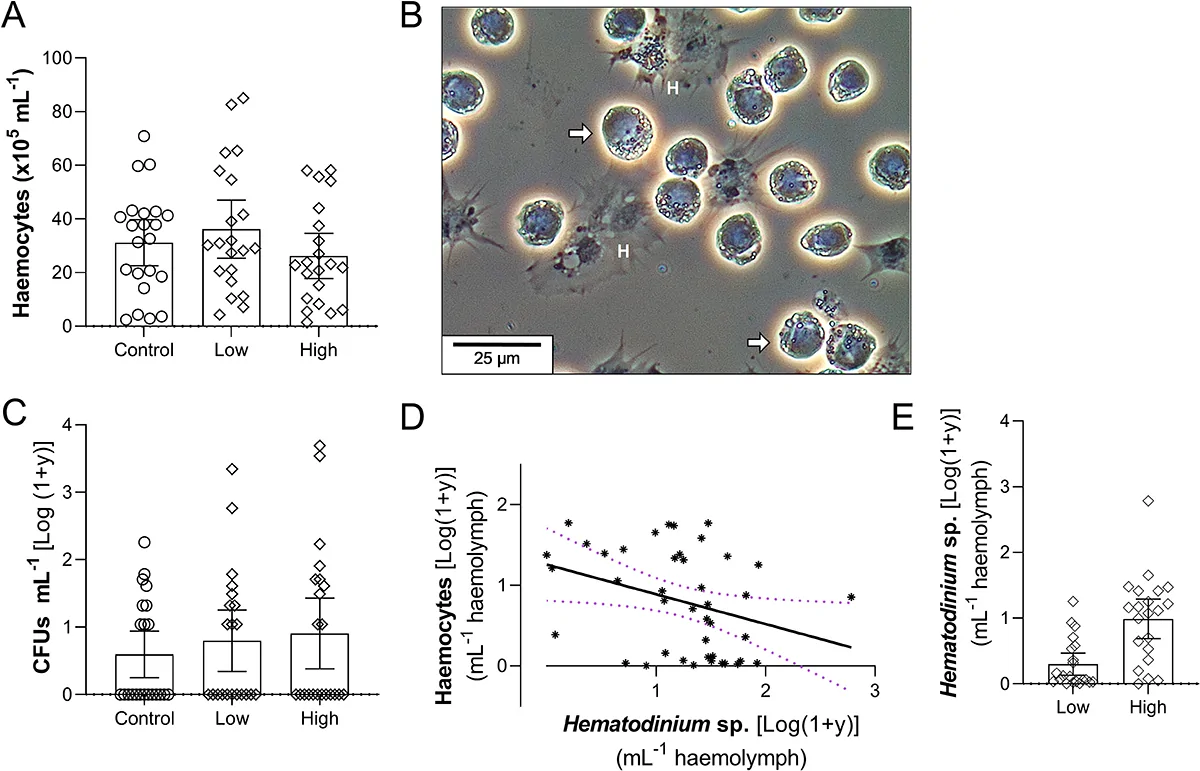
Hemolymph factors of *Hematodinium* sp. infected shore crabs. (A) Total hemocyte counts of shore crabs in the absence (control) and presence of *Hematodinium* sp. with low and high parasite loads (*n* = 66 in total). (B) phase contrast micrograph of shore crab hemolymph depicting *Hematodinium* sp. spheroid trophonts (unlabeled white arrows) alongside host hemocytes (H). (C) numbers of culturable bacterial colony forming units (CFUs) in the hemolymph of control and parasitized crabs. A general growth medium, TSA supplemented with 2% NaCl, was used. (D) correlation of hemocyte and *Hematodinium* sp. numbers in the hemolymph of parasitized crabs. A linear regression was fitted, with 95% confidence intervals (C.I.). (E) total numbers of *Hematodinium* sp. in the hemolymph of crabs with “low” and “high” *Hematodinium* sp. loads. In all figures, data are depicted as mean values with 95% C.I. Data in panels C, D and E were log transformed [Y = log(y + 1)].

**Figure 2.**
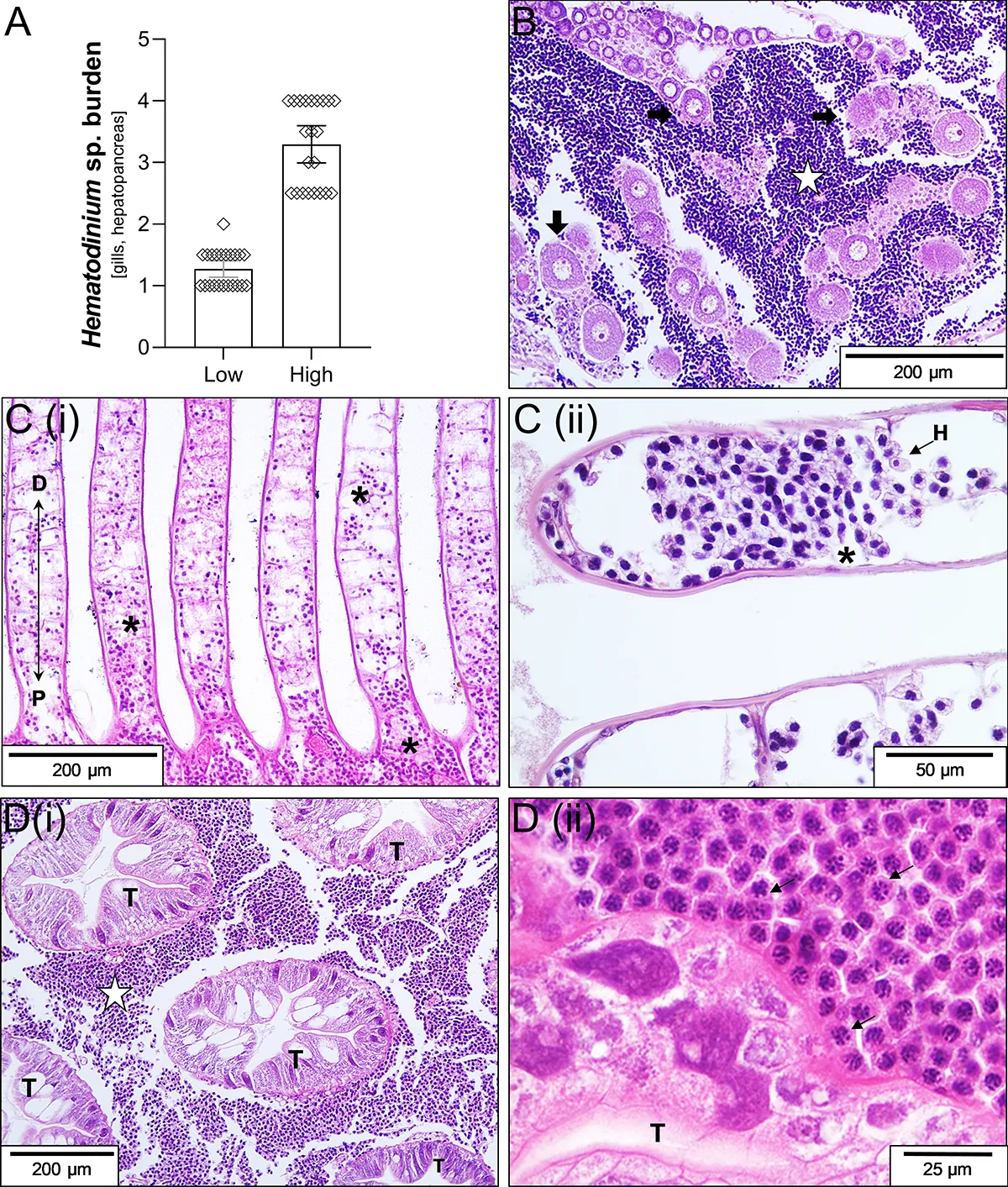
Histopathological condition of *Hematodinium* sp. infected shore crabs. (A) levels of *Hematodinium* sp. in the solid tissues of shore crabs quantified into “low (0.5 < 2)” and “high (2.5 < 4)” categories. Tissue sections of gills and hepatopancreas from individual crabs were graded on a scale of 0 (no parasite) to 4 (gross burden), then averaged. (B) female gonadal tissue occupied by accumulating *Hematodinium* sp. (white star) and oocytes (black arrows). C(i) and C(ii) low and high power micrographs, respectively, of *Hematodinium* sp. in gill tissue. Asterisks (*) denote parasite masses/clumps. Distal (D) and proximal (P) directions are indicated. H, host hemocytes. D(i) and D(ii) low and high power micrographs, respectively, of *Hematodinium* sp. in hepatopancreatic tissue. The inter-tubule space is replete (white star) with *Hematodinium* sp. T, tubule. Condensed dinokaryon nuclei of *Hematodinium* sp. ameboid trophonts (unlabelled black arrows) are visible.

Herein, to investigate putative dysbiosis associated with *Hematodinium* sp. infection, a total of 66 *C. maenas* (out of the archived samples) were scrutinized based on several characteristics, notably the gross parasite burden (control, low, high) of liquid and solid tissues (Figure 1 and 2) in patently infected crabs and “matched” across key variables: season (rather than month), location, sex, carapace color (indicator of time post-molt), and width (size). Twenty-two crabs were considered highly (clinical, terminal) infected based on average intensity scores of 2.5 ≤ 4 (see Table 1). To ensure parity among sample groups, 22 crabs with low intensity scores of 0.5 ≤ 2 (based on average gill/hepatopancreas histology) were selected. Control crabs (*n* = 22) were only designated “*Hematodinium*-negative” when all diagnostic tools – haemocytometry, PCR assays, multi-tissue histology – failed to show *Hematodinium* sp. presence (Table 1; Figure 1; Figure 2).

**Table 1.** Shore crab biometrics and *Hematodinium* sp. infection status in hemolymph and solid tissues.

Group	Carapace width (mm)	Fouling^a^	Limb loss^a^	Hemolymph appearance	Hemocyctes^b^ (x10^5^ mL^−1^)	*Hematodinium* sp. PCR	*Hematodinium*^b^ sp. (x10^5^ mL^−1^)	*Hematodinium* sp. load^c^ (Gills, Hepatopancreas)	CFUs^b^ (mL^−1^)
Control (*n* = 22)	50.5 ± 2.2	5 / 22	8 / 22	1 / 22 = milky	31.2 ± 4.1	0%	0	0	44 ± 18 (*n* = 9/22)
Low (*n* = 22)	52.5 ± 2.5	4 / 22	5 / 22	4 / 22 = milky	36.2 ± 5.2	100% positive	2.13 ± 0.88	1.3 ± 0.33	299 ± 219 (*n* = 10/21)
High (*n* = 22)	48.7 ± 1.7	7 / 22	7 / 22	7 / 22 = milky	26.2 ± 4	100% positive	41.1 ± 28.5	3.3 ± 0.15	881 ± 561 (*n* = 10/22)

a = presence/absence of fouling (epibionts) on carapace; one or more missing limbs.

b = numbers of hemocytes/*Hematodinium*/bacteria per ml hemolymph.

c = based on grading criteria (0 = no parasites in either tissue, 4 = tissue entirely congested with parasites).

### High-throughput sequencing and bioinformatic processing of the shore crab microbiome

All 66 hemolymph samples were sent for metabarcoding alongside an extraction blank generated in parallel using the same Qiagen Blood and Tissue Kit. Samples were prepared for sequencing with Eurofins Genomics (Germany) using their established INVIEW Microbiome Profiling 3.0 service; each consisting of a 25 μL extract with ~50 ng DNA per μL. One amplicon per crab sample was generated using a two-step PCR protocol followed by Illumina paired end sequencing (MiSeq, 2 × 300bp) focussing on the V3-V4 hypervariable region of the bacterial 16S rRNA gene using the following primers: Fwd: TACGGGAGGCAGCAG [44], Rev: CCAGGGTATCTAATCC [45]. Sequence libraries were prepared and processed using an optimized in-house protocol consisting of standard Illumina adapter sequences and Illumina technology. Two samples failed, one each from the parasitized groups, yielding 64 quality microbiomes.

Bioinformatic analyses were performed by Eurofins Genomics according to the following protocols, and unless otherwise specified, all processing was performed using in-house (Eurofins) scripts. Demultiplexing of reads according to index sequences was performed on all reads that passed the standard Illumina chastity filter (PF reads). This was followed by primer clipping in which forward and reverse primer sequences were removed from raw reads. Primer clipping settings were defined as to not allow any mismatches within the primer sequences and read pairs containing mismatched primer sequences were removed. Read merging was performed according to the algorithms within FLASH (2.2.0 [46]), which consider all possible overlaps and creates consensus sequences based on the highest quality value. Reads were merged when the ends of the forward and reverse sequences overlapped to provide longer stretches of the target region. When merging was not possible due to read length, the forward read was retained for processing. Reads were further processed by quality filtering in which merged reads significantly shorter or significantly longer than expected minimal/maximal target region length were discarded, as well as reads containing ambiguous bases (“N”). All reads retained following these processing steps were used for microbiome profiling.

The first step of the microbiome profiling analysis was to identify and remove chimeric reads using the *de-novo* algorithm of UCHIME [47] within the VSEARCH package [48]. The remaining reads were processed using minimum entropy decomposition (MED [49]; and [50]) to partition marker gene datasets into Operational Taxonomic Units (OTUs). This method allows for a decomposition of sequence data sets with a single nucleotide resolution and also identifies and filters “noise” within the dataset, i.e. very low abundance sequences. DC-Megablast was used for taxonomic assignment for each OTU (Internal reference database: /dbdir/nt.filtered.fa), with the minimum criteria of sequence identity of 70% across at least 80% of the representative sequences [51]. Additional processing of OTUs was performed using QIIME software (version 1.9.1, http://qiime.org/ [52]) using default settings, e.g. 97% threshold for OTU picking. Taxonomic units with less than 0.1% reads were collapsed into “Other” and OTUs for which no database match could be assigned were considered “Unclassified” (Supplementary Table 2).

OTU “background correction.” All samples were manually filtered to remove signals of contamination as identified by reads present in the extraction blank. Briefly, the read number of each OTU in the extraction blank was subtracted from the number in the respective sample to give a new read abundance. If the value was lower than the number present in the extraction blank then this became 0, if for example, the read number in the extraction blank was 200, this was subtracted from the number in the sample, e.g. 300, to give a remaining total of 100. Any OTUs that were only present within the extraction blank were removed from the OTU table and subsequent downstream analysis. Sample B1 (Crab extraction blank) contained 138 OTUs, 86 of which were unique to the blank sample. A further 13 OTUs were removed from the data set as following processing were no longer present in any sample. In total 99 OTUs were removed from the data set. OTUs that remained in the data set (*n* = 39) were filtered for contamination based on numbers in the extraction blank. Once filtering had been performed, the extraction blank was removed from the OTU table and not included in any downstream analysis. In order to further “clean” the data set, any OTUs that corresponded to eukaryotes (68) were removed from the dataset prior to downstream analysis. This included the removal of OTUs assigned to Bacillariophyta, Chlorophyta, Rhodophyta, and Streptophyta i.e. diatoms, algae, grasses, ferns, mosses. A signal for *Paravannella minima* (183 reads), assigned to one sample was also removed from the data set. There were 142 OTUs assigned as “Unclassified” corresponding to 1.63% of the total remaining OTUs following cleaning (Supplementary Table 2).

### Defining the core bacteriome

To identify core bacterial taxa that were consistently associated with different *Hematodinium* sp. intensity levels, we built the core bacteriome across samples from the same intensity levels by applying strict filters to the OTU sample compositional data. We followed the rationale to build the core microbiome developed by Astudillo-García et al. [53] but varying the fraction of samples used to define OTU persistence. In particular, we defined the core bacteriome for each disease intensity level independently as the set of OTUs found in samples belonging to the corresponding intensity level and which were (i) present with a relative abundance of at least 0.01% across the entire dataset and (ii) found in at least some fraction of the samples belonging to the corresponding disease intensity level, which we varied across 66% (i.e. 2/3^rds^ as in [53] 50%, 40% and 30%. We used different thresholds for OTU persistence across samples within disease levels because the more stringent threshold (2/3^rds^) kept only one OTU across the whole dataset. Hence, we relaxed this constraint to obtain a more comprehensive representation of the core bacteriome. It is worth noting that this suggests that the set of OTUs in the core bacteriome is sensitive to the specific persistence threshold used to define it. Furthermore, since the core bacteriome defined at the OTU level comprised a limited number of taxa across all threshold values used (see Results), we also defined the core bacteriome at the genus level using the same procedure described above but only focusing on the 2/3 and 50% threshold levels. To obtain a representative number of read counts at the genus level (i.e. avoid biases generated by the skewed distributions of reads across OTUs), we took the median value of read counts across OTUs belonging to each genera. We standardized the read counts by dividing the resulting genus-level number of reads by their sum across each sample.

### Data handling and statistical analyses

Our study adheres to the ARRIVE guidelines (see Supplementary Online Checklist). Cellular data – hemocyte counts, CFUs and *Hematodinium* sp. per mL hemolymph – as well as gill and hepatopancreas parasite levels (graded 1 to 4) were analyzed and visualized in GraphPad PRISM v9. Data were checked for Gaussian distribution (normality) using the D’Agostino & Pearson test, and if necessary, log transformed [Y = log(y + 1)] prior to Unpaired t-tests (or Mann-Whitney) and one‑way ANOVA (or Kruskal Wallis). Additionally, Spearman rank/correlation tests (and linear regression) were used to assess the putative relationships between *Hematodinium* sp. in the hemolymph and the numbers of hemocytes and CFUs. Data are presented as the mean with 95% confidence intervals in figures and mean ± standard error in text. Initial microbiome-related data analyses were performed in R using default and in-built settings in the phyloseq package (http://joey711.github.io/phyloseq/index.html), notably for calculating alpha-indices (AEC Chao1, Fisher, InverseSimpson, and Shannon).

To quantify differences in community structure across samples, ordination analysis on community data was conducted using non-metric multidimensional scaling (NMDS) and Bray-Curtis dissimilarity to calculate pairwise distances between samples. Hellinger transformation (square root of relative abundances) was applied to the OUT table, a matrix with samples as rows and bacterial operational taxonomic units (OTUs) as columns with the elements of the matrix representing the number of reads found in each sample per OTU, prior to ordination to reduce skewness and better reflect the contribution of each OTU to their corresponding bacteriome. To facilitate visualization, points in the ordination plots were grouped (and colored accordingly) based on the explanatory variables explored in this analysis: parasite/disease level (control, low, high), sampling location, season and biological sex of the sampled individuals. To quantify variability within groups, we calculated ellipses centered at the centroid of each group and representing the standard deviation across samples. Data transformation, NMDS ordination, and calculation of ellipses were performed using the functions decostand, *metaMDS* and *ordiellipse* from the vegan package in R respectively [54]. For NMDS ordination we used 3 dimensions in order to improve stress by setting the parameter k = 3 on the *metaMDS* function.

To assess the influence of infection intensity, sampling location, season, and biological sex of sampled individuals on the differences observed across the sampled bacteriomes we conducted permutational multivariate analysis of variance (PERMANOVA) on the Hellinger transformed data using the factors mentioned above as explanatory variables. We further explored pairwise differences between groups within each of the factors explored individually using pairwise PERMANOVAs. PERMANOVA and pairwise PERMANOVA analyses were actioned using the functions *adonis2* in vegan [54] and *pairwise.adonis2* in the *pairwiseAdonis* [55] R package, respectively.

### Putative metabolic signatures

To explore the differences in the functional profiles of the bacteriome of control vs high- and low-diseased crabs we inferred the potential repertoire of metabolic functions associated with each of the OTUs present in the bacteriomes of the different disease intensity levels (control, low, high). OTU metabolic profiles were obtained based on the Kyoto Encyclopedia of Genes and Genomes (KEGG) Ontology (KO) using Tax4Fun2 [56]. Tax4Fun2 uses complete genomes available through the KEGG database and their associated functional profiles to generate a database of reference functional profiles against which the taxonomic classifications of given OTUs at the genus level are matched. In this way available OTUs from our dataset are assigned a specific metabolic profile. In the event that a taxonomic classification has no match in the functional references, the corresponding OTU is not included in the metabolic prediction. Relative abundances of the matched OTUs against the reference functional profiles per sample are used to weight the functions associated to each sample. The sample-level profile thus obtained is subsequently normalized, resulting in the sum of all functions in a sample = 1.

To assess the differences between functional profiles across disease intensity levels (control, high, low) we conducted pairwise PERMANOVA analyses on the log-transformed functional profiles of each sample using intensity level as a predictor variable as done above for bacteriome composition. Ordination was also performed using NMDS as above with dimensions k = 3 to improve the measure of stress. Further, to identify functions that were significantly different across treatments of levels of disease intensity, we modeled relative abundances of functions across samples against intensity level. We fit generalized linear models with negative binomial response to multivariate functional data against intensity (i.e. ($f_{1},f_{2},f_{3},\ldots ,f_{n}$) ~ factor(*IntensityLevel*); where $f_{i}$ is the relative abundance of function i in the sample. On the model outputs we used resampling-based analysis of deviance (with 999 bootstrapped iterations) to make function-specific inferences about their associations with specific disease intensity levels. Significance level (unadjusted *p-value*) was calculated for each response variable (i.e. each function) and a threshold of 0.01 (i.e. *p-value* < 0.01) was used to select the functions that were statistically significantly associated with each treatment levels. Multivariate generalized linear models were fit using *manyglm* and deviance analyses were performed using the *anova.manyglm* functions of the *mvabund* package in R [57], respectively. Relative abundances of the functions thus selected per intensity level were plotted in a heatmap using the *pheatmap* function of the *pheatmap* package in R.

## Results

### Hemolymph parameters of healthy and Hematodinium sp. infected shore crabs

Total hemocyte (immune cell) counts varied between the three sample groups, with *Hematodinium*-positive crabs fluctuating by ±5 × 10^5^ mL^−1^ (Figure 1(A,B)) when compared to the *Hematodinium*-negative control (F_(2, 61)_ = 1.225, *p* = 0.301). Notably, there was a strong inverse correlation between hemocyte and *Hematodinium* sp. numbers within the parasitized animals (Spearman *r* = −0.4009 (95% C.I. −0.6334 to −0.101, *p* = 0.0085; Figure 1(D)). Culturable bacterial CFUs were detected in 41% of *Hematodinium*-negative crabs and ≥45% in *Hematodinium*-positive crabs (Table 1). For control crabs, this equated to 44 ± 18 CFUs mL^−1^ (*n* = 9), increasing to 299 ± 219 and 881 ± 561 CFUs mL^−1^ for crabs with low (*n* = 10) and high (*n* = 10), respectively (Table 1). There was an apparent marked increase in bacterial CFUs in hemolymph from parasitized crabs, however, the difference was non-significant (F_(2, 62)_ = 0.544, *p* = 0.583; Figure 1(C)). Moreover, although there was an apparent positive correlation between the numbers of *Hematodinium* and bacterial CFUs in the hemolymph, it was non-significant (Supplementary Figure S1).

Numbers of *Hematodinium* sp. trophonts in the liquid tissue (hemolymph; Figure 1(D,E)) for “low” and “high” level infection groups reflected the disease intensity scores of their solid tissues (gills and hepatopancreas; Figure 2(A). In the hemolymph, parasite levels differed by 19-fold (Figure (1E); Mann-Whitney, *p* = 0.0003) and >2.5-fold in the gill/hepatopancreas (Figures 2(A); Mann-Whitney, *p* < 0.0001) – clearly distinguishing crabs with low vs high infection status. At the terminal stage of disease, tissues such as the gonads (Figure 2(B)), gills (Figure 2(C)) and hepatopancreas (Figure 2(D)) were replete with trophonts of *Hematodinium* sp.

### Hemolymph bacteriomes differ between healthy and Hematodinium sp. infected shore crabs

Following sequencing, we secured a final shore crab microbiome dataset of 64 samples split across *Hematodinium*-negative controls (*n* = 22) and two *Hematodinium*-positive groups, low (early clinical, *n* = 21) and high (terminal clinical, *n* = 21). Samples were derived from 31 female and 33 male crabs in the final data set (Supplementary Table 3). In total, we retrieved 2,011,666 reads across the 64 samples (median = 26,942; mean = 31,432; min. 13,482 to max. 180,325), which represented 8,731 taxa from 24 phyla (Supplementary Figure 2).

**Figure 3.**
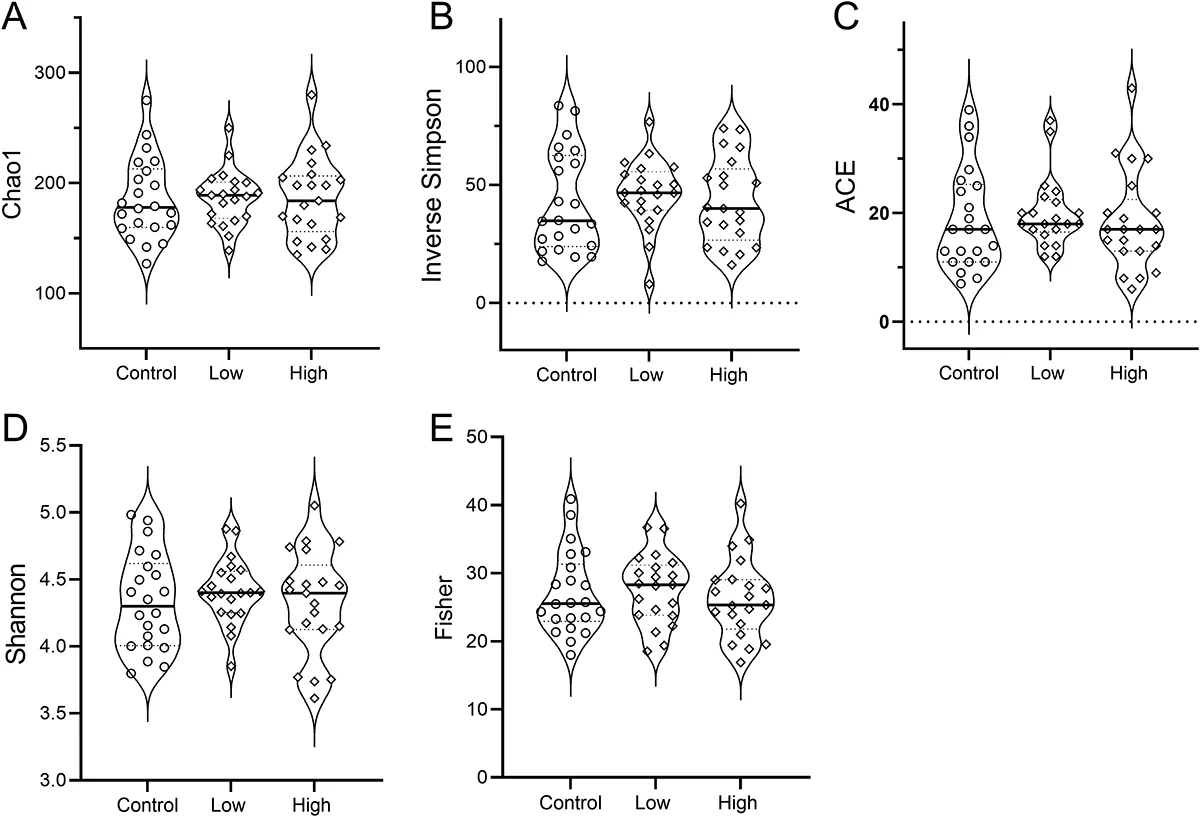
Alpha-diversity indices of the hemolymph bacteriome in healthy and *Hematodinium* sp. infected shore crabs, *Carcinus maenas*. A) Chao1, B) InverseSimpson, C) ACE, D) Shannon, and E) Fisher. Data are formatted as violin plots with median values drawn as continuous black lines, and first/third quartiles indicated by broken black lines. n = 22 (control) and n = 21 each for low and high *Hematodinium* sp. intensities.

According to alpha-diversity indices (Shannon, Chao1, Fisher, ACE, InverseSimpson), there were no remarkable changes in the hemolymph bacteriomes of control and *Hematodinium*-positive shore crabs (Supplementary Figure 3), even when sub-categorized by intensity (Table 2; Figure 3). Crabs with low levels of *Hematodinium* sp. contained a marginally more rich/diverse hemolymph bacteriome. For example, median species richness (Chao1) values for control, low and high parasite levels, were 178, 189, and 184, respectively. The most prominent change was in species diversity (InverseSimpson), with the median value increasing 34% from control to low *Hematodinium* levels, and 15% from control to high *Hematodinium* levels (Figure 3), though each pairwise comparison was non-significant (*p* > 0.05; Table 2).

**Table 2.** Alpha-diversity indices for the hemolymph bacteriome of healthy and *Hematodinium*-positive shore crabs, *Carcinus maenas*. Median values are expressed with mean +/- standard error in parentheses. *p*-values are expressed to two decimal places.

Index	Control	*Hematodinium*-positive		Pairwise comparisons (*p*-values)		
		Low	High	Control vs Low	Control vs High	Low vs High
AEC	17 (18.77 ± 1.98)	18 (19.94 ± 1.4)	17 (18.43 ± 1.98)	*p* = 0.30	*p* = 0.97	*p* = 0.25
Chao1	178 (185.7 ± 7.8)	189 (186.9 ± 5.4)	184 (186.6 ± 7.9)	*p* = 0.64	*p* = 0.94	*p* = 0.93
Fisher	25.5 (27.3 ± 1.29)	28.3 (27.62 ± 1.11)	25.3 (26.22 ± 1.27)	*p* = 0.52	*p* = 0.73	*p* = 0.32
Inverse Simpson	34.8 (42.93 ± 4.53)	46.7 (46.15 ± 3.17)	40 (43.08 ± 3.94)	*p* = 0.35	*p* = 0.82	*p* = 0.42
Shannon	4.3 (4.33 ± 0.08)	4.4 (4.41 ± 0.05)	4.4 (4.32 ± 0.08)	*p* = 0.33	*p* = 0.97	*p* = 0.55

**Figure 4.**
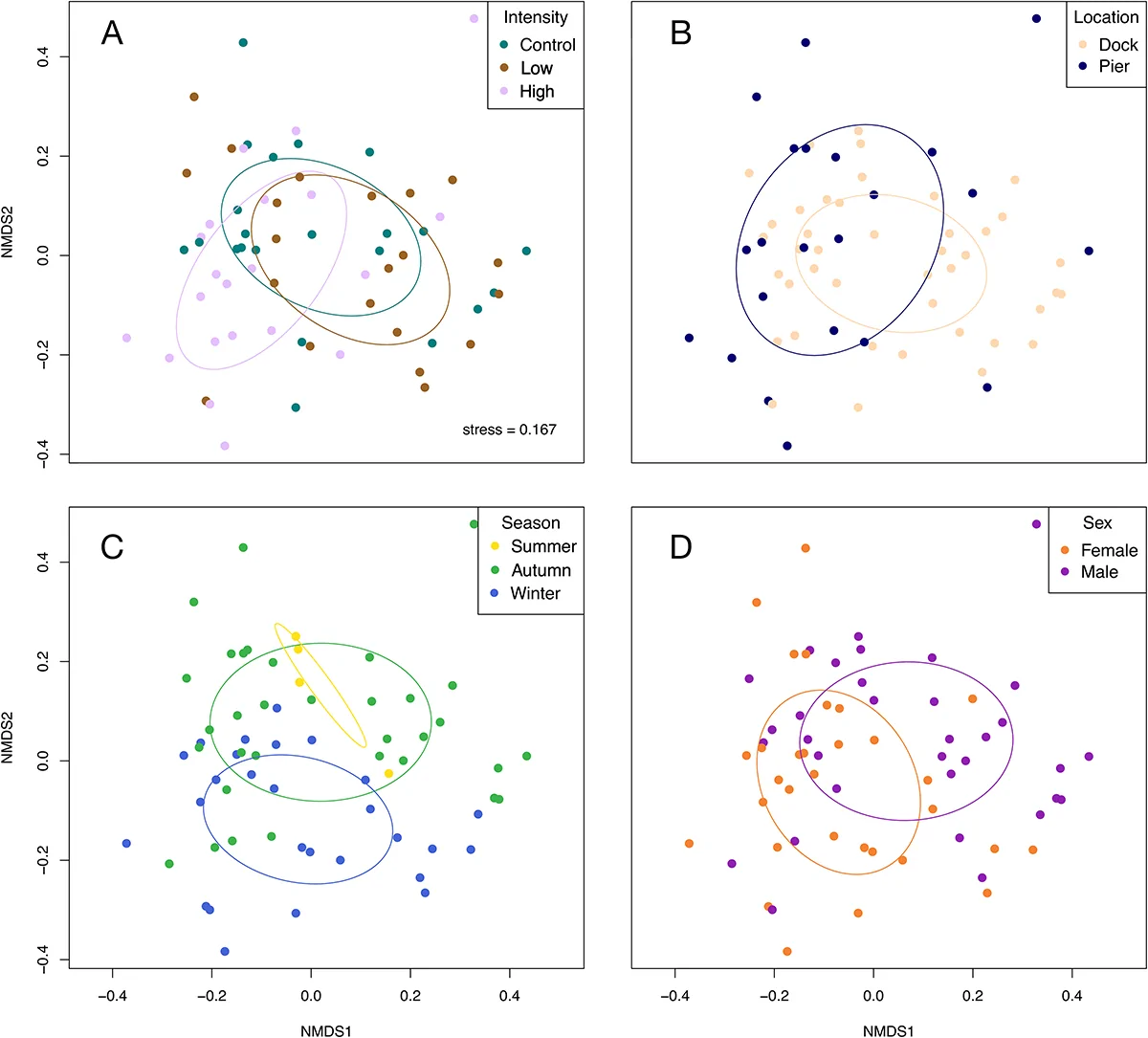
Community structure of the bacteriome in the shore crab (*Carcinus maenas*) is influenced by environment, biology and *Hematodinium* sp. intensity. Non-metric multidimensional scaling ordination of the bacterial communities found in the shore crab (*Carcinus maenas*) based on Bray-Curtis distance of the Hellinger-transformed number of reads of the Operational Taxonomic Units (OTUs) found in each sample. Each point represents a sequenced sample (16S rRNA) from an individual crab specimen. Across panels, points and ellipses (representing the spread of the points in the form of the standard deviation of the mean / centroid of each group) are coloured by the corresponding factor: (A) infection intensity, (B) sampling location, (C) season, and (D) biological sex of the specimens.

Overall, we found differences in the structure of the shore crab bacteriome driven by a combination of environmental and biological factors (Figure 4, Table 3). Multivariate analysis of the variance across samples from different sites (dock vs pier) and *Hematodinium* sp. intensity (control, low, high) revealed that all the factors analyzed here (including biological sex) significantly influenced the differences in structure of the bacterial communities across sampled individuals, even if the variance explained by the joint effect of these factors was 12% (Table 3). Moreover, when looking at each factor in isolation, we found the bacteriomes of crabs with high *Hematodinium* sp. loads were different from those of both low and control individuals, even if the extent of this difference between high and control was marginal (Figure 4, PERMANOVA: High vs Low F = 1.30, *p* = 0.006; High vs Control F = 1.12, *p* = 0.063). Differences in the bacterial communities between control individuals and those with a low infection intensity were clearly non-significant (PERMANOVA: F = 0.98, *p* = 0.518). Sampling location, season and biological sex of the specimens all influenced significantly the differences across bacteriomes (Figure 4A-4D); Supplementary Table S4).

**Table 3.** Environment, biology, and *Hematodinium* sp. intensity influence the structure of the hemolymph bacteriome. Results from the multivariate analysis of variance test comparing the differences in crab bacterial communities in relation to season, sampling location, biological sex, and infection intensity. (DoF = degrees of freedom, SumOfSqs = sum of squares, F = F statistic of the ANOVA model, *p* = value of statistical significance).

	DoF	SumOfSqs	R^2^	F	*p*
Disease Intensity	2	1.05	0.04	1.16	0.013
Location	1	0.68	0.02	1.49	0.001
Sex	1	0.53	0.02	1.17	0.026
Season	2	1.13	0.04	1.24	0.003
Residual	57	25.93	0.88		
Total	63	29.33	1

**Figure 5.**
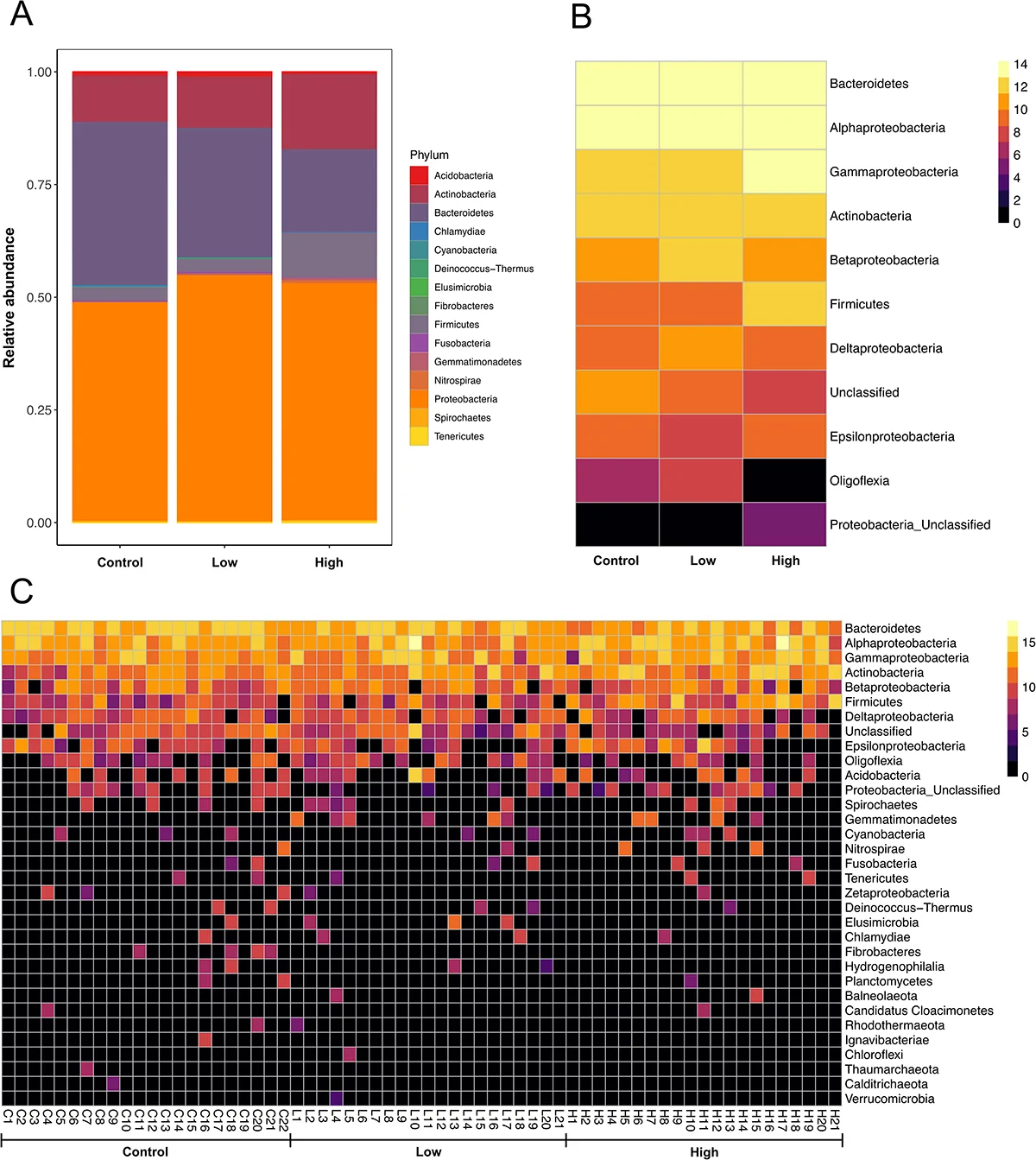
Taxonomic profiles of the shore crab bacteriome vary across levels of *Hematodinium* sp. (disease) intensity. (A) relative abundances of the 15 most abundant phyla across control (i.e. no disease), low and high disease intensity. (B) taxonomic profile represented as the log10-transformed median of the number of counts across samples belonging each disease intensity levels. (C) phylum and subphylum level representation (rows) of taxonomic composition across “C” control, “L” low intensity and “H” high intensity samples (columns). Colors represent log10-transformed number of counts per sample at the corresponding taxonomic resolution. For panel (C), the non-bacterial thaumarchaeota are represented in a single animal from the control (*Hematodinium*-negative) group.

For all three groups, Proteobacteria dominated at ~50–55% of the bacterial population, followed by Bacteroidetes (~19–37%), Actinobacteria (~10–17%), Firmicutes (up to 10%) and Acidobacteria (≤2%; Figure 5(A)). As the *Hematodinium* sp. infection progressed, Bacteroidetes representation dwindled from ~37% in the control to <30% at the early/low phase, to <20% during parasitemia; partly attributed to a gross reduction in bacteria within the Class Flavobacteriia (Supplementary Figure 2). Concurrently, Actinobacteria and Firmicutes representation increased from ~10% to ~19%, and from ~3% to 10%, respectively (Figure 5(A,B)). When reviewing phyla and classes in each crab, Bacteroidetes, α- and γ-Proteobacteria and Actinobacteria were invariable, followed by Firmicutes found in 94% (60/64) of microbiomes (Figure 5(C)).

**Figure 6.**
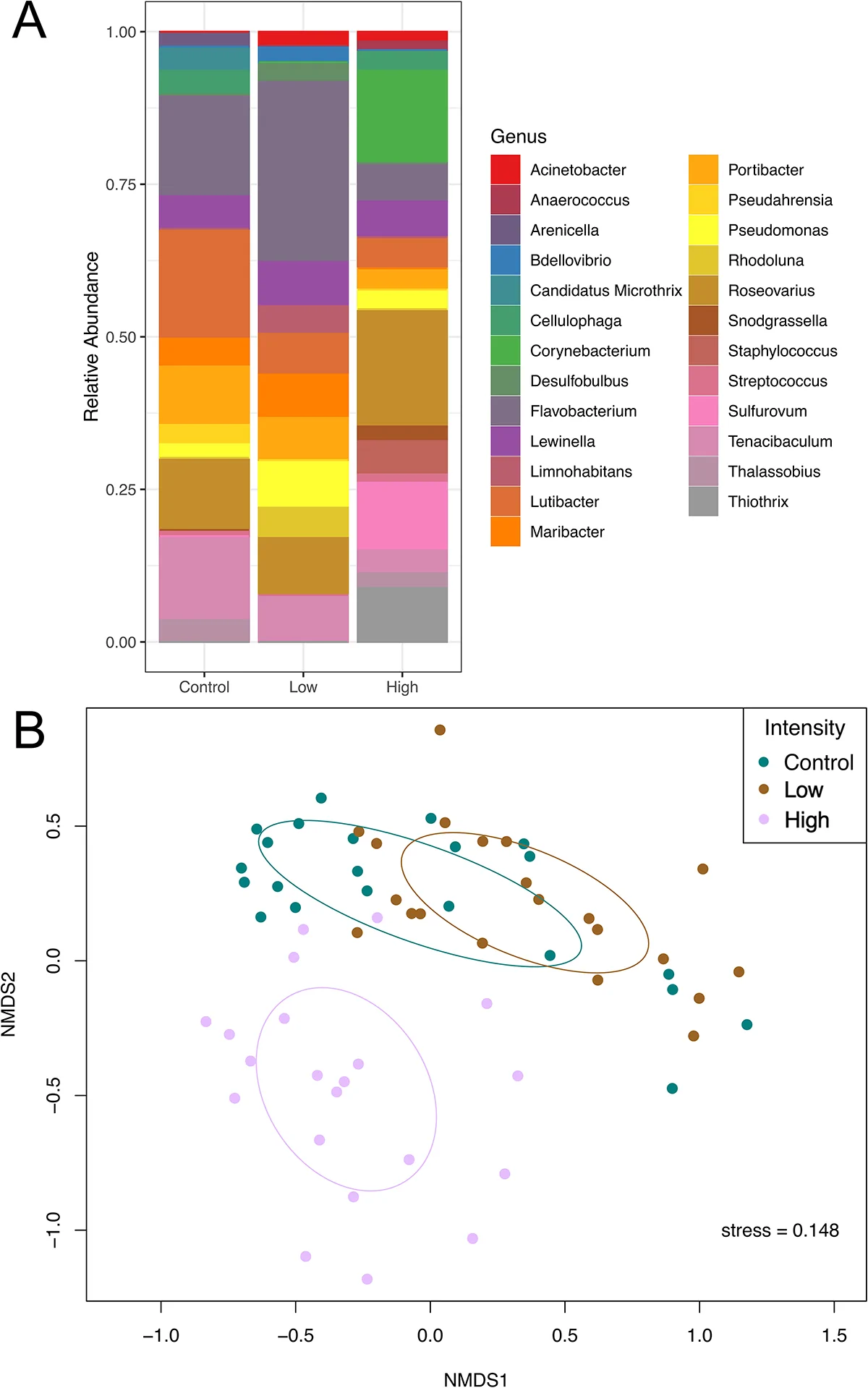
Genus-level core bacteriome of shore crabs changes during *Hematodinium* sp. disease progression. (A) relative abundances of core bacteriome genera across disease intensity levels (control, low and high). (B) non-metric multidimensional scaling ordination of the core bacteriome (OTUs found in at least 50% of sample per disease intensity level and with relative abundances > 0.01% across the entire dataset) aggregated to the genus level found in the shore crab (*Carcinus maenas*) based on Bray-Curtis distance of the Hellinger-transformed median number of reads of the operational taxonomic units (OTUs) from the same genus found in each sample. Each point represents a sequenced sample (16S rRNA) from an individual crab specimen.

We observed a high level of inter-individual variation within the crab hemolymph bacteriome, making it challenging to define core bacteriomes among individual animals with the same *Hematodinium* sp. intensity (Supplementary Figure 2; Supplementary Table 5). Using the commonly adopted threshold for OTU persistence in core microbiomes of 2/3^rds^ of the samples, only one OTU was selected across the whole dataset, whereas a 50% fraction of sample threshold yielded nine core OTUs (see Supplementary Table 5 for other core definitions). To dig deeper into the components of the core bacteriome, we proceeded to extract core members at a higher level of taxonomic resolution: Genus. Aggregation of OTUs to the genus level resulted in 764 unique taxa. Building the core microbiome at 66% threshold prevalence at the level of *Hematodinium* sp. intensity (i.e. control, low, high) yielded seven genera across the entire dataset, however, using a 50% threshold resulted in a set of 25 genera out of the 764 (Figure 6(A)). The genus level core bacteriome unveiled strong differences in community composition (Figure 6(B)), with core genus-level bacteriomes being significantly different across *Hematodinium-*positive and negative groups (Figure 6(B), pairwise PERMANOVA: Control vs Low F = 7.04, *p* < 0.001; Control vs High F = 9.95, *p* < 0.001; Low vs High F = 13.6, *p* < 0.001).

### Bacteriome metabolic profiles in healthy and Hematodinium sp. infected shore crabs

Overall, our metabolic search returned 8,439 hits to metabolic functions from an average of 43.3% (s.d. = 16.7%) of the OTUs present across the bacteriomes of all sampled individuals, with the most striking differences in functional profiles across disease intensity levels being observed between the two *Hematodinium*-positive groups (PERMANOVA: High vs Low F = 1.7283, *p* = 0.034; Supplementary Figure S4).

**Figure 7.**
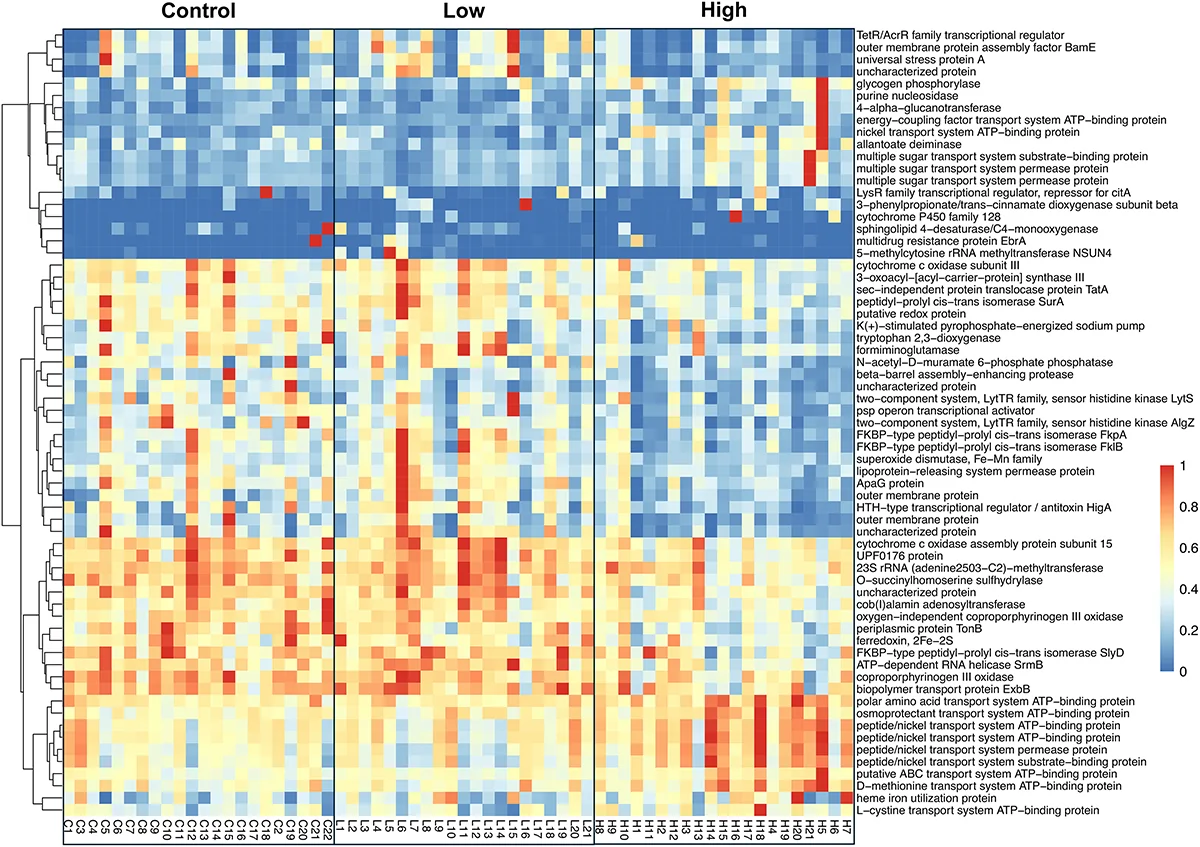
Predicted metabolic signatures of the hemolymph bacteriome between control and *Hematodinium*-positive shore crabs, *Carcinus maenas*. Data on functional metabolic signatures (obtained through OTU putative metabolic function assignment using Tax4Fun2) are presented as a heatmap filtered using a significance value of *p* = 0.002 as the threshold for metabolic function assignment per sample from a multivariate generalized linear model with disease intensity level as a predictor variable (see Methods). Rows are ordered by disease/*Hematodinium* intensity (control – low – high), whereas columns are ordered from left to right based on the abundance of the functions.

To narrow our focus into the main functional differences across groups, we ran bootstrapped analysis of deviance on multi-variate abundance analysis of the functional data using *Hematodinium* intensity (control, low, high) as a predictor variable to determine which of those 8,439 hits were significantly different across groups. This analysis yielded 283 metabolic functions that were more expressed within some disease intensity levels than others (*p* < 0.01; Supplementary Figure (5A)). Factors associated with nutrition, cell stress responses, and virulence dominated the functional profiles. While there is some variation across the individual samples, bacteriomes isolated from crabs with advanced (high) hematodiniosis were distinct from those with low intensity infections and those absent *Hematodinium* (control; Supplementary Figure (5A). For the former, enhanced representation of protein-related catabolism was highlighted including tissue breakdown (e.g. the polyamines putrescine and spermidine) as well as stress/damage management: proline iminopeptidase, transport system ATP-binding proteins and permeases for oligopeptides, individual amino acids (e.g. cysteine), nitrates/sulfonates (NitT/TauT) and osmoprotectants, as well as heme export (protein A) and utilization (Supplementary Figure (5B); Figure 7). Concurrently, there were blocks of metabolic functions under-represented in the high‑intensity *Hematodinium* group compared to the low‑intensity *Hematodinium* group associated with oxidative stress, carbohydrate/fatty acid metabolism, spore formation and virulence: stringent starvation proteins (A & B), tellurium resistance proteins (TerD, TerZ), superoxide dismutase (Fe-Mn), heavy metal resistance (CzcA), isocitrate lyase, cell division, cell cycle and biofilm-related proteins (FtsB, FTsL, ZapD, DivJ, RpfC, LadS), lipopolysaccharide assembly protein B, glycosyltransferases, spore maturation proteins (A & B), FKBP-type peptidyl-prolyl cis-trans isomerases (FkpA, FkIB), and insecticidal complex toxin protein Tcc (Supplementary Figure (5B), Figure 7).

When applying an even more stringent threshold of 0.002, 65 metabolic functions were differentially represented among the disease/parasite intensity levels (*p* < 0.002; Figure 7). Factors involved in the uptake of amino acids and small peptides, such as peptide/nickel transport system ATP binding and permease proteins, polar amino acid and _D_-methionine transport system ATP-binding proteins, were retained and remained distinguishable across *Hematodinium* intensity levels (Figure 7). Comparing low to high *Hematodinium* intensities, the bacteriome stress response signatures were preserved as well as proteins linked to biofilm formation and virulence (e.g. TetR/AcrR, superoxide dismutase, antitoxin HigA, LytS, alginate-related AlgZ).

## Discussion

### Biotic and abiotic factors shaping the hemolymph microbiome of shore crabs, Carcinus maenas

Tissues of decapod crustaceans, including the hepatopancreas, gut, and hemolymph, are neither sterile nor axenic. Reports of bacteremia in seemingly healthy crustaceans – showing no clinical signs of disease – go back decades. According to Tubiash et al. [58], over 80% of wild blue crabs (*Callinectes sapidus*) from Chincoteague Bay (Virginia, USA) contained a mean bacterial load of 1,876 CFUs per mL hemolymph (*n* = 290). Male crabs had twice as many bacteria as females, and hemolymph bacterial density peaked during the summer months. Scott and Thune [59] sampled 1,560 red swamp crayfish (*Procambarus clarkii*) from commercial ponds and isolated bacteria from 41% of the cohort. Bacterial numbers were elevated alongside water temperature (>24°C) and chlorophyll a content. For kuruma prawns (*Marsupenaeus japonicus*) held under culture conditions, Wang and Wang [60] quantified 10^4^-10^7^ CFUs/g in solid tissues (e.g. stomach) and 10–1000 CFUs/mL hemolymph.

The microbiomes of decapod crustaceans, like other animals, contribute to health, nutrition, and biological defenses [37,61,62], whereas excessive growth or disturbance due to pathogens or noninfectious stressors can lead to disease (e.g. pathobiome [35]). Using a nonselective growth medium, tryptone soy agar (+2% salt), we found around four in ten shore crabs contained culturable bacterial colony forming units (CFU), with increased numbers trending with *Hematodinium* sp. intensity (albeit non-significantly). Culture-dependent methods for bacterial identification and quantification are limited to taxa that grow moderately to non-fastidiously under aerobic (or facultative) conditions and are prone to false negative results (e.g. [63,64]). Ooi et al. [65] used both culture-dependent (Zobell marine agar) and independent (high throughput sequencing, HTS) approaches to screen spiny lobster (*Panulirus ornatus*) hemolymph for bacterial presence. Bacteria belonging to the family Rhodobacteraceae were well-represented using either approach, however, HTS revealed higher bacterial loads, diversity, and relative abundances. Using 16S rRNA meta-barcoding, we profiled the hemolymph microbiome of the shore crab *Carcinus maenas* to reveal a bacterial consortium dominated by Proteobacteria, followed by Bacteroidetes, Actinobacteria, Firmicutes, and Acidobacteria (in descending order). These phyla, alongside Fusobacteriota, are found routinely in microbial profiles of decapod crustacean hemolymph, e.g. *C. sapidus* and *Penaeus vannamei* [62,66], though their abundances vary. For example, Chinese mitten crab *Eriocheir sinensis*, contained ~69% Proteobacteria, ~15% Bacteroidota, ~6% Firmicutes, and >2% Actinobacteria, with various known (e.g. Desulfobacterota) and unknown taxa forming the remaining population [67]. According to Martin et al. [21], Proteobacteria similarly dominated *Hematodinium*-free *N. norvegicus* hemolymph at ~66%, followed by Firmicutes (~20%), Bacteroidetes (~10%), and Actinobacteria (>1%).

Environmental heterogeneity and host characteristics shape microbiome assemblage, diversity, and function. In a meta-analysis performed by Cornejo-Granados et al. [66] covering 199 microbiomes from 16 studies, salinity (marine vs freshwater) was the dominant environmental driver of bacterial composition. Sample origin (wild, laboratory, farmed), host developmental stage (adult, juvenile, larval), tissue location, diet, and disease status all affected the alpha-diversity measures of bacteria. For shore crabs studied herein, sampling location (pier, dock), time of year (Summer, Autumn, Winter) and biological sex (male, female) influenced the hemolymph bacteriome to measurable extents. In a yearlong experiment, Holt et al. [39] tracked the microbiomes of European lobsters (*H. gammarus*) reared in either a sea-based container culture system or land-based culture system, reporting a more species rich and diverse gut bacteriome for the former. Lobster age as well as infection status (absence/presence of a nudivirus, HgNV) were additional contributing factors. Martin et al. [40] concluded that sampling point (March vs June) was the main driver of variation in bacterial composition of the velvet crab (*Necora puber*) hepatopancreas, with no discernible impact of the microparasite *Paramarteilia canceri*.

Shore crabs, brown crabs, and langoustine all show seasonal peaks and troughs in *Hematodinium* sp. burden and differential susceptibility to parasitism between males and females [14]. Small male shore crabs in the Spring/Summer are the most likely demographic to be infected with *Hematodinium* sp. [68]. Through interrogation of bacterial beta-diversity and establishing a core microbiome at genus level, we differentiated the subtle contribution of *Hematodinium* sp. presence from host and environmental factors.

### Progression of Hematodinium sp. infection and hemolymph dysbiosis in shore crabs, Carcinus maenas

**Figure 8.**
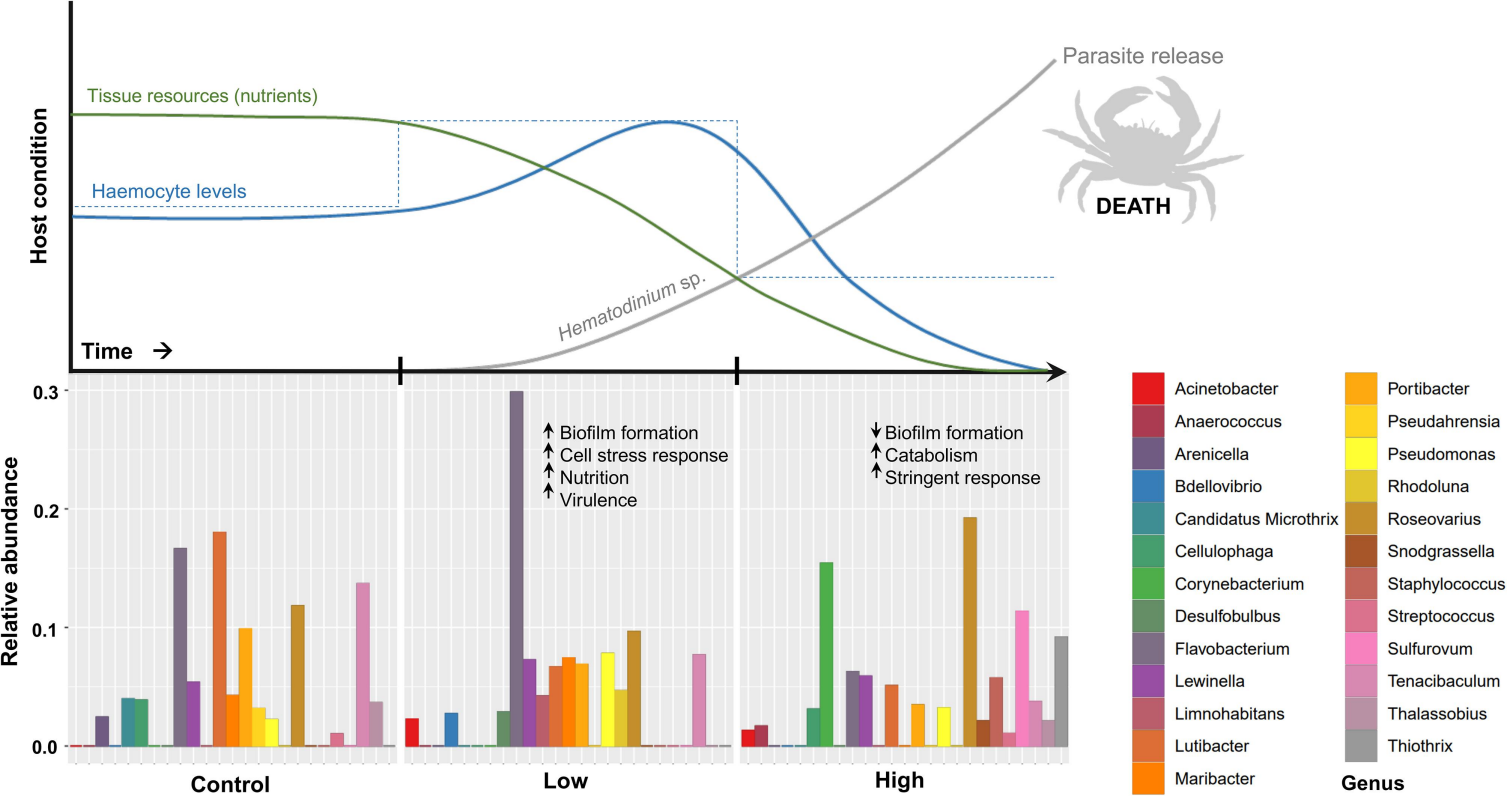
Progression of *Hematodinium* sp. infection in shore crabs. Upper panel: schematic representation of host-deterioration over time focussing on hemolymph condition. The blue lines represent hemocyte levels from this study according to control, low and high (broken lines), and a general trend from other crab sources (continuous line). The gray line represents *Hematodinium* sp. (trophont) numbers. The green line represents tissue resources, freely accessible in the hemolymph (e.g. glucose, protein), and those stored in the hepatopancreas (e.g. glycogen). Lower panel: relative abundances of core bacteriome genera across control (absent *Hematodinium* sp.), low and high groups; data were harvested from Figure 6. Taxonomic profiles represent the log_10_-transformed median of the number of counts across samples. Inset for the “low” and “high” categories are the most striking differential metabolic signatures (reduced or enhanced) for each group (data were harvested from Figure 7). Data on functional metabolic signatures were obtained through OTU putative metabolic function assignment using Tax4Fun2.

We did not detect substantive changes in hemolymph alpha-diversity (bacterial richness, diversity, or evenness) in the presence of *Hematodinium* sp. in shore crabs. Conversely, Martin et al. [21] reported reduced bacterial richness (Chao1) in the hemolymph (but not the gut) of *Hematodinium-*positive langoustine, which was apparent in subclinical (PCR-positive) cases, and persisted into clinical, late-stage infection. Reduced alpha-diversity measures of tissue bacteriomes are often represented in studies of diseased and stressed crustaceans, examples include nudivirus-infected lobsters (*H. gammarus* [39]); sulfamethoxazole-exposed signal crayfish (*Pacifastacus leniusculus* [69]); white spot syndrome virus-infected swamp crayfish (*P. clarkii* [70]). We did, however, find compositional changes, i.e. differential abundances of taxa, coinciding with disease progression from control to low to high *Hematodinium* sp. intensity. There was a spike (3-fold increase) in putative pathobionts, e.g. Class Flavobacteriia, from control to low *Hematodinium* levels, which was reflected in metabolic signatures associated with virulence and biofilm formation. As *Hematodinium* sp. accumulate in the hemolymph, *Flavobacterium* diminish (Figure 8). The metabolic profiles we obtained for *Hematodinium* intensity groups (control, low, high) differed most noticeably for stress management and nutrition. As the infection progresses to parasitemia and the crabs are succumbing to *Hematodinium* sp. (i.e. through the loss of hemocytes and presence of devitalized tissue; Figure 8), there was a transition in the bacteriome from a virulence-related profile (e.g. detoxification, spore formation) to nutrition and consolidation (e.g. protein catabolism, cell protection).

We discovered changes in bacteriome composition and function in the core hemolymph bacteriome attributed to *Hematodinium* sp. Differential abundance of several genera became more pronounced with *Hematodinium* sp. intensity. Enhanced representation of *Roseovarius, Sulfurovum* and *Thiothrix* spp. (alpha-, gamma- and epsilon-proteobacteria, respectively), as well as *Corynebacterium* spp. (Actinobacteria) distinguished shore crabs with the greatest *Hematodinium* sp. burden. The latter genus contains some species that are pathogenic to fish (e.g. striped bass [71]), though have been noted in crustacean hemolymph previously (e.g. American lobsters, *Homarus americanus* [72]). Some *Roseovarius* species, e.g. *R. litoreus*, have been used as a probiotic for swimming crab *Portunus trituberculatus* [73], while others like *R. crassostreae* cause disease outbreaks (juvenile oyster disease) in eastern oysters *Crassostrea virginica* [74]. Interestingly, known pathogenic taxa *Pseudomonas* and *Flavobacterium* are conspicuous in the low *Hematodinium* intensity group, which is in stark contrast to their diminished representation in the high intensity group. This taxonomic shift in bacteria mirrors the gross shift in metabolic profiles from virulence (biofilm formation) and stress/toxin‑related factors to amino acid utilization (catabolism). Bacterial genera like *Sulfurovum* and *Thiothrix* oxidize sulphur and this may explain, in part, their emergence in shore crabs with high *Hematodinium* sp. burden and switch in metabolic functions to peptide and amino acid binding/permeases. Accumulation of sulphur through tissue and protein decomposition, notably those rich in cysteine and methionine, is associated with tissue putrefaction and anaerobiosis. Enhanced representation of transport system ATP-binding proteins specific to _D_-methionine and _L_-cysteine are highlighted in the metabolic functions of bacteria from heavily parasitized shore crabs. Such bacteria can have a symbiotic role in some crustaceans in extreme environments (e.g. hydrothermal vents [75]), though the latter has been linked to filamentous bacterial disease in crustaceans alongside pathogens like *Leucothrix* and *Beggiatoa* spp (e.g. penaeids [76]).

It is worth highlighting the scarcity of *Vibrio* spp. in the shore crab hemolymph bacteriome – regardless of *Hematodinium* sp. status. *Vibrio* spp. are numerous in the marine environment, and representatives cause major diseases in decapod crustaceans (e.g. limp lobster disease, acute hepatopancreatic necrosis disease [77,78]). Herein, the genus *Vibrio* appears within the top 20 (at number 15), though it is not retained in the core microbiome analysis for control or *Hematodinium* sp. infected crabs (Figure 8). Using end-point PCR, we previously detected *Vibrio* spp. presence in the hemolymph and surrounding waters of shore crabs [20] in Swansea Bay (Wales, UK). These data suggest that *Vibrio* spp. are commensal in shore crabs, but are not a dominant member of the microbiome. Alternatively, shore crabs may not be susceptible to pathogenic vibrios in these waters.

### The shore crab-Hematodinium sp. pathosystem

Previously, we confirmed the enzootic status of *Hematodinium* sp. within *C. maenas* populations of Swansea Bay, and discriminated host (sex, size) and environmental (location, seasonal) factors associated with parasitization [42], including seasonal variability in disease incidence and severity. We gained further insight into *Hematodinium*-related health decline in shore crabs, specifically co-infection occurrence and hemocyte reactivity toward the parasite [20]. Parasite/pathogen profiles of shore crabs can be distinguished based on location across Swansea Bay, and herein, we extend this pattern to their resident bacteriome. *Hematodinium*-positive shore crabs were no more likely to harbor co-infections – fungal, bacterial, or micro/macro-parasitic – than *Hematodinium*-free individuals. Crucially, tissue micrographs depicted host hemocytes responding to other pathogen- and damage-associated molecular patterns independent of *Hematodinium* presence, and, when isolated *Hematodinium* trophonts were exposed *in vitro* to donor crab hemocytes, no immune-reactions were recorded. Beyond shore crabs, a lack of immune reactivity toward *Hematodinium* sp. extends to crustaceans from temperate waters, e.g. edible (brown) crabs [27], and Norway lobsters [21]. Histological inspection of *N. norvegicus* tissues, notably the gut, did not contain evidence of hemocyte reactivity toward *Hematodinium* (even in heavily infected individuals where parasite trophonts occluded hemocoel spaces leading to tissue rupture), and no visible co-infections were encountered [21]. Apparent immune-modulation in shore crabs may be due to interference with hemocyte communication, as we quantified significantly fewer extracellular vesicles during parasitemia (~70% reduction [22]), and post-translational modification (citrullination) of cellular proteins, actin and Dscam. In the *Hematodinium perezi* – *Portunus trituberculatus* pathosystem, immune-suppression was proposed to occur via several mechanisms, including diminished pathogen recognition and hemocyte phagocytic capacity [23,24].

*Hematodinium* sp. infection in shore crabs is not an acutely fatal disease; instead, it is chronic and pernicious. This strategy, in part, avoids triggering cellular immune reactivity and the selection of aggressive pathobionts emerging from the resident microbiome. There exist intricate links and balance between host microbiota and the innate immune system – decapod crustaceans are no exception [60,62]. The resident microbiota could assist the host by competing with would-be pathogens, and maintaining basal immunity levels. All things considered, we cannot rule out a syntrophic relationship between the parasitic dinoflagellate and the shore crab microbiome. Several key biosynthetic pathways are absent in the *Hematodinium* genome, e.g. isoprenoids (non-mevalonate or DOXP pathway and the canonical mevalonate or MVA pathway [79]), therefore, they must source these from their host. As nutrient levels diminish, the host seems to lack the ability to allocate resources rather than acquire nutrients. To the best of our knowledge, there are no reports of decreased feeding efforts in *Hematodinium-*parasitized crustaceans; in fact, infected *N. norvegicus* leave their burrows twice as much as *Hematodinium*-negative individuals in what Stentiford et al. [80] considered was to forage for more food and/or oxygen to compensate for the parasite’s impact on nutrition and respiration. Additionally, Martin et al. [21] observed the stomach contents of *Hematodinium* sp. infected *N. norvegicus* as being either full or partially filled. Parasitized crustaceans are usually lethargic, e.g. reduced escape/swimming behaviors [81], and depleted of nutrient stores (e.g. protein, glucose, and glycogen [25,26].

We postulate that the hemolymph bacteriome of *Hematodinium*-infected shore crabs reflects the deteriorating host condition, specifically the transition from metabolic derangement to exhaustion. As the growing parasite horde raids the available nutrients, the resident bacteria (commensals, mutualists, or opportunistic pathogens) respond to the metabolic deficit (Figure 8).

## Supplementary Material

Clean Copy of Supplementary Material - QVIR-2026-0028.R1.docx

## Data Availability

All data are available immediately and have been included in the original text, online supplementary material, BioProject PRJNA1406313 (https://www.ncbi.nlm.nih.gov/bioproject/1406313), and REF68 https://doi.org/10.6084/m9.figshare.31056004].
