# Supplementary material for: Hematodinium sp. infection alters hemolymph bacteriome composition and function in shore crabs, Carcinus maenas
Source: Virulence. 2026 Jul 26;17(1):2709259. doi: 10.1080/21505594.2026.2709259 (PMC13501986; doi:10.1080/21505594.2026.2709259)
Supplement: Supplementary file 1 [file KVIR_A_2709259_SM5259.docx]

**Supplementary Table 1. PCR-based detection of *Hematodinium* spp.**

Each assay was performed in a T100 PCR thermal cycler (BioRad Laboratories Inc., Hemel Hempstead, UK).

|  | Primers (forward, reverse) | Cycling conditions | Target size | Reference |
| --- | --- | --- | --- | --- |
| Assay 1 | 0.5 µM (equimolar):  **Hemat-F-1487** (CCTGGCTCGATAGAGTTG),  **Hemat-R-1654** (GGCTGCCGTCCGAATTATTCAC) | Initial denaturation of 94°C (10 min), followed by 30 cycles of 94°C (15s), 54°C (15s), 72°C (30s), and final elongation at 72°C (10 min) | 187 bp | Gruebl et al. (2002) |
| Assay 2 | 1 µM (equimolar):  **18SF2** (CAGTTTCTGGAAGTGGCAGCTG),  **Hem3R** (TAACCCGAGCCGAGGCATTCA) | Initial denaturation of 94°C (1 min), followed by 35 cycles of 94°C (1 min), 58°C (1 min), 72°C (1 min), and final elongation at 72°C (10 min) | 480 bp | Small et al. (2006); Eigemann et al. (2010) |

- Gruebl T, Frischer M, Sheppard M, Neumann M, Maurer A, Lee R. Development of an *18S* rRNA gene targeted PCR based diagnostic for the blue crab parasite *Hematodinium* sp. Dis Aquat Org. 2002;49:61–70.
- Small HJ, Neil DM, Taylor AC, Atkinson RJ, Coombs GH. Molecular detection of *Hematodinium* spp. in Norway lobster *Nephrops norvegicus* and other crustaceans. Dis Aquat Org. 2006;69:185–95.
- Eigemann F, Burmeister A, Skovgaard A. *Hematodinium* sp. (Alveolata, Syndinea) detected in marine decapod crustaceans from waters of Denmark and Greenland. Dis Aquat Org. 2010;92:59–68.

**Supplementary Table 2. Sequence reads and OTU assignment summary.**

| Total number of input sequences | 6 981 386 | 100.00% |
| --- | --- | --- |
| Remaining sequences after pre-processing and quality filtering | 6 980 123 | 99.98% |
| Remaining sequences after chimera detection and filtering | 6 967 924 | 99.81% |
| Total number of sequences assigned to OTUs | 5 414 650 | 77.56% |
| Total number of sequences assigned to taxa | 5 339 785 | 76.49% |
| Copy-number corrected total count | 2 115 601 | - |
| Total number of OTUs (before ‘cleaning’, i.e., before blank contaminants and eukaryotes were removed) | 8898 | 100.00% |
| Number of OTUs assigned to taxa | 8756 | 98.40% |
| Number of OTUs following removal of ‘blank contamination’ | 8799 | 98.89% |
| Number of OTUs following removal of ‘Eukaryota’ | 8731 | 98.12% |
| Number of Unclassified OTUs | 142 | 1.63% |

**Supplementary Table 3. Details on crab samples from microbiome assessments: site, sex and season.**

|  | **n** | **Dock** | **Pier** | **Male** | **Female** |
| --- | --- | --- | --- | --- | --- |
| Overall | 64 | 42 | 22 | 33 | 31 |
| Control | 22 | 14 | 8 | 13 | 9 |
| Low | 21 | 15 | 6 | 11 | 10 |
| High | 21 | 13 | 8 | 9 | 12 |

|  | **n** | **Autumn** | **Winter** | **Summer** |
| --- | --- | --- | --- | --- |
| Overall | 64 | 33 | 27 | 4 |
| Control | 22 | 13 | 8 | 1 |
| Low | 21 | 9 | 10 | 2 |
| High | 21 | 11 | 9 | 1 |

**Supplementary Table 4. Pairwise statistical tests of the differences between bacterial communities in shore crabs across environmental, biological and infection conditions.** Results from pairwise permutational analyses of variances (PERMANOVA) tests comparing each of the levels of the four factors considered here as potential drivers of the structure of the bacteriome in shore crabs (*Carcinus maenas*): infection intensity, sampling location, season and biological sex of the specimens. (DoF = Degrees of Freedom, SumOfSqs = Sum of Squares, F = F statistic of the ANOVA model, p = p value of statistical significance).

|  | **DoF** | **SumOfSqs** | **R^2^** | **F** | **p** |
| --- | --- | --- | --- | --- | --- |
| ***Disease Intensity*** |  |  |  |  |  |
| Control vs. Low | 1 | 0.45 | 0.02 | 0.98 | 0.518 |
| Control vs. High | 1 | 0.52 | 0.03 | 1.12 | 0.063 |
| High vs Low | 1 | 0.61 | 0.03 | 1.30 | 0.006 |
|  |  |  |  |  |  |
| ***Sampling Location*** |  |  |  |  |  |

| Dock vs Pier | 1 | 0.69 | 0.02 | 1.49 | 0.001 |
| --- | --- | --- | --- | --- | --- |
|  |  |  |  |  |  |
| ***Season*** |  |  |  |  |  |
| Autumn vs. Summer | 1 | 0.52 | 0.03 | 1.13 | 0.067 |
| Autumn vs. Winter | 1 | 0.63 | 0.02 | 1.36 | 0.003 |
| Winter vs. Summer | 1 | 0.60 | 0.04 | 1.31 | 0.006 |
|  |  |  |  |  |  |
| ***Biological sex*** |  |  |  |  |  |
| Male vs. Female | 1 | 0.57 | 0.02 | 1.22 | 0.017 |

**Supplementary Table 5. Relationship between core microbiome threshold and number of OTUs remaining.**

| **Fraction of samples threshold** | **Number of OTUs in core** |
| --- | --- |
| 66% | 1 |
| 50% | 9 |
| 40% | 22 |
| 30% | 57 |

**Supplementary Figure 1.** Numbers of bacterial colony forming units (CFUs) versus *Hematodinium* sp. in the haemolymph of shore crabs. Data (n = 42 per variable) were log transformed [Y=log(y+1)]. Linear regression line representing the mean and 95% confidence intervals.

A Spearman rank test was performed, yielding a positive, though non-significant, correlation between the numbers of CFUs and microparasites per mL.

Spearman r = 0.1672 [95% CI, -0.1532 to 0.4557], P = 0.2900

**Supplementary Figure 2. Phylum (A), Class (B, C) and Genus-level (D) representation (abundance) within the haemolymph bacteriome of control and *Hematodinium*-positive shore crabs**. C = control, 1-22; L = low parasite burden, 1-21; H = high parasite burden, 1-21. Twenty-four phyla were identified.

**Supplementary Figure 3. Alpha-diversity indices for control and *Hematodinium*-positive shore crabs, *Carcinus maenas.* A)** Shannon; **B)** Chao1; **C)** Fisher; **D)** InverseSimpson; **E)** ACE. Data are formatted as violin plots with statistical comparisons (p-values). Control (*Hematodinium*-negative; blue) versus *Hematodinium-*positive (green) crabs. In this instance, data from both low and high *Hematodinium* sp. groups were combined into a single category.

**Supplementary Figure 4. Gross structure of the shore crab (*Carcinus maenas*) bacteriome according to putative metabolic functions.** Non-metric multidimensional scaling ordination of the bacterial communities found in the shore crab (*Carcinus maenas*) based on Bray-Curtis distance of metabolic hits for each sample.

**Supplementary Figure 5A. All 263 differentially expressed predicted metabolic signatures of haemolymph bacteriomes in control and *Hematodinium*-positive shore crabs.**

**Supplementary Figure 5B. Metabolic signatures/modules that differ between control and low *Hematodinium* levels versus high *Hematodinium* levels.**
